# Epistasis between antibiotic resistance mutations and genetic background shape the fitness effect of resistance across species of *Pseudomonas*

**DOI:** 10.1098/rspb.2016.0151

**Published:** 2016-05-11

**Authors:** T. Vogwill, M. Kojadinovic, R. C. MacLean

**Affiliations:** 1 Department of Zoology, University of Oxford, Oxford, UK; 2 Université Aix-Marseille–CNRS, Marseille, France

**Keywords:** antibiotic resistance, fitness costs, genetic background, *Pseudomonas*

## Abstract

Antibiotic resistance often evolves by mutations at conserved sites in essential genes, resulting in parallel molecular evolution between divergent bacterial strains and species. Whether these resistance mutations are having parallel effects on fitness across bacterial taxa, however, is unclear. This is an important point to address, because the fitness effects of resistance mutations play a key role in the spread and maintenance of resistance in pathogen populations. We address this idea by measuring the fitness effect of a collection of rifampicin resistance mutations in the β subunit of RNA polymerase (*rpoB*) across eight strains that span the diversity of the genus *Pseudomonas*. We find that almost 50% of *rpoB* mutations have background-dependent fitness costs, demonstrating that epistatic interactions between *rpoB* and the rest of the genome are common. Moreover, epistasis is typically strong, and it is the dominant genetic determinant of the cost of resistance mutations. To investigate the functional basis of epistasis, and because *rpoB* plays a central role in transcription, we measured the effects of common *rpoB* mutations on transcriptional efficiency across three strains of *Pseudomonas*. Transcriptional efficiency correlates strongly to fitness across strains, and epistasis arises because individual *rpoB* mutations have differential effects on transcriptional efficiency in different genetic backgrounds.

## 1. Background

Antibiotic resistance is associated with pleiotropic costs that are expressed in terms of reduced competitive fitness in the absence of antibiotics [1–3]. Fitness costs play a key role in the dynamics of resistance as they generate selection against resistance in antibiotic-free conditions, such as when antibiotic use is discontinued, or during transmission between hosts. Understanding the factors that govern the cost of resistance is therefore crucial for predicting when resistance will persist in pathogen populations. Put simply, the larger the cost associated with resistance, the less likely the persistence of resistance in bacterial populations over the long term.

Most antibiotics target highly conserved domains in proteins that play pivotal roles in cell biology, such as DNA replication, cell-wall assembly and protein synthesis [4]. The most direct mechanism for bacteria to evolve resistance to antibiotics is by altering the structure of these targets [5]. Because the target sites of antibiotics are highly conserved, target site mutations show a strong tendency to evolve in parallel across species of bacteria [6–8]. This parallel molecular evolution implies that these mutations have some similar phenotypic effects in these differing bacterial species, in that these mutations increase resistance to antibiotics. However, the extent to which the fitness costs of resistance mutations are conserved across genetic backgrounds remains unclear (but see [6]). There is growing evidence that mutations often have epistatic effects on fitness in bacteria [9–12], suggesting that genetic background could play a key role in shaping the cost of resistance across species.

In this paper, we investigate the influence of genetic background on the fitness cost of antibiotic resistance, using a collection of rifampicin-resistant mutants from the genus *Pseudomonas* as a model system [7]. One advantage of working with *Pseudomonas* is that the genus is highly diverse, and yet it is still possible to culture most strains under a common set of laboratory conditions [13,14], making it possible to obtain equivalent measures of fitness in different species or strains of bacteria. Rifampicin is an antibiotic which binds to a highly conserved domain of RNA polymerase, preventing RNA-transcript elongation. Resistance to rifampicin evolves by mutations in *rpoB* that alter the structure of the rifampicin binding pocket. The fitness cost of rifampicin resistance has been measured across a wide range of bacteria, including *Escherichia coli* [15], *Salmonella typhymurium* [6], *Pseudomonas aeruginosa* [16], *Mycobacterium tuberculosis* [17] and *Staphylococcus aureus* [18]. However, previous studies have measured fitness using a range of techniques and under different environmental conditions [3,19]. As these variables can affect the estimate of the cost of resistance, it is questionable to solely rely on comparing fitness cost estimates from different studies.

To measure the overall contribution of genetic background to the cost of resistance, we estimated the competitive fitness of a collection of mutations across eight strains that span the diversity of *Pseudomonas*. We then did two further analyses to try to explain why certain mutations vary in their fitness effects between genetic backgrounds. First, we use comparative methods to test whether the effects of a mutation are more conserved between closely related strains. If genetic background is a significant but relatively minor determinant of the cost of a mutation, we would predict a greater probability of differing fitness effects with increasing genetic distance. Second, rifampicin resistance mutations are known to have a range of effects on transcription [15], and previous work has shown that the fitness effects of rifampicin resistance correlate with reductions in transcriptional efficiency [15,20]. Genetic background could influence the fitness effects of rifampicin resistance mutations by modulating the effect of altered RNA polymerase on transcriptional efficiency or by altering the strength of the correlation between transcriptional efficiency and fitness. In other words, genetic background can influence the fitness effects of mutations by altering the relationship between genotype and phenotype, or the relationship between phenotype and fitness. To address this issue, we used an inducible reporter construct to measure the transcriptional efficiency of a subset of rifampicin resistance mutations across three strains of *Pseudomonas*.

## 2. Material and methods

### (a) Strains and culture conditions

Eight strains of bacteria from the genus *Pseudomonas* were used: *Pseudomonas aeruginosa PAO1, P. stutzeri ATCC17588, P. mendocina CCUG7181, P fulva CCUG12573, P. putida KT2440, P. protegens PF5*, *P. fluorescens PF01* and *P. fluorescens SBW25.* Prior to experimentation, all strains were stored at −80°C in 25% glycerol. All culturing was performed at 30°C with constant shaking at 200 r.p.m., in King's B (KB) medium.

### (b) Isolation of rifampicin-resistant mutants

Rifampicin-resistant mutants were obtained from Vogwill *et al.* [7] where they were isolated by fluctuation tests on rifampicin agar. Briefly, for each strain, an overnight culture was diluted 1 million-fold and used to found 480 parallel cultures. These were grown for 48 h before being plated on agar containing either 30 or 60 mg ml^−1^ of rifampicin. After 48 h, mutants were isolated from 93 independent cultures and frozen in 25% glycerol at −80°C. We then sequenced the two regions of *rpoB* that can result in high-level rifampicin resistance. A single example of each mutation by strain combination was selected for further analysis.

### (c) Measuring the cost of resistance

To facilitate the competition experiments, we transformed the ancestral genotype of each strain with a chromosomally integrated green fluorescent protein (GFP). These strains were generated by integrating a constitutively expressed GFP marker at the chromosomal tn7 insertion site using the methods of Choi & Schweizer [21]. Fitness costs were measured by competing rifampicin-resistant mutants against the appropriate GFP-tagged rifampicin-sensitive ancestral strain. Competitions took place in 200 µl of KB medium in a 96-well plate, incubated at 30°C with constant shaking at 250 r.p.m. Competitions lasted 24 h. Each competition was replicated six times, with the replicates of each competition spread across at least two separate occasions. For each competition, cells were grown overnight in KB medium. Each mutant was then mixed 50 : 50 by volume with the GFP-tagged ancestor, and this mixture was then diluted 10 000-fold. Initial and final ratios of GFP-tagged to untagged cells were determined using BD C6 flow cytometer. 10 000 cells per culture were counted and scored as either fluorescently tagged or not. Fitness was calculated at the ratio of the number of doublings of the rifampicin-resistant mutant compared with the GFP-tagged ancestral strain. To control for the cost of GFP-expression, fitness was standardized relative to the fitness of the unmarked ancestor in competition with the GFP-tagged ancestor.

### (d) Correcting for phylogenetic distance

Using a selection of strains from across a genus results in potentially confounding any results with the effects of phylogeny. Specifically, the hierarchical nature of most phylogenies results in not all tips of a phylogeny being equally independent from each other. For example, in a phylogeny of three species, unless the phylogeny is a star configuration, two strains must be more closely related to each other than to the third strain. If a trait has any phylogenetic correlation or bias, then it would be pseudo-replication to consider all three strains equally independent. The method of Felsenstein [22] overcomes this issue by first comparing the phenotypes of the two closely related species, and then comparing the phenotype of the third species with the average of the first two species.

The analysis across phylogenies can be further conflated as not all evolutionary distances within a phylogeny are likely to be equal. This latter point could have important consequences for the influence of genetic background on the effects of a mutation, as it would seem intuitive that differing fitness effects are less likely between closely related strains. To control for this, we correct for the topological effects of phylogeny using the method of Felsenstein [22]. However, as we wished to test the effects of evolutionary distance on the effects of a mutation, we do not standardize our contrasts by evolutionary distance as in the conventional Felsenstein approach. We would therefore expect a positive correlation between evolutionary distance and the size of a contrast, assuming the probability of a mutation having conserved effects is greater between closely related strains.

The phylogeny of this study system has been previously determined (see [7] for full methods), based on 55 housekeeping genes taken from 28 fully sequenced *Pseudomonas* genomes. It is a maximum-likelihood phylogenetic tree assuming a GTR + G + I model in PHYML [23]. From this phylogeny, we trimmed it to just the eight strains used here, from which we extracted the relevant evolutionary distances for each pair of strains, or a strain and a node, or two nodes, as appropriate.

### (e) Measuring gene expression per mutant

To estimate the effect of the *rpoB* mutations on transcriptional efficiency, we use the luciferase report gene system to measure gene expression [20]. In bacteria, transcription and translation are strongly coupled; therefore, gene expression should provide a good proxy for transcriptional efficiency. To measure the effects of *rpoB* mutations on transcriptional efficiency, strains were transformed with an IPTG-inducible luciferase reported gene (henceforth lux-transformed). The luciferase gene, which reacts with ATP to produce light, is commonly used to measure transcriptional activity by measuring the amount of light produced per cell. This was performed for the three most genetic tractable strains, specifically the ancestral strain for *P. aeruginosa PAO1, P. putida KT2440* and *P. fulva CCUG12573,* as well as seven mutations common to these three strains. For each strain, overnight cultures of the lux-transformed ancestor and each of the lux-transformed mutants were grown in KB medium. Cultures were then diluted 10 000-fold into 200 µl of KB medium in black 96-well Costar microplates with clear bottom (Corning, USA). The medium was supplemented with 1 mM IPTG to induce luciferase production. The plate was then incubated in a Synergy 2 microplate reader (BioTek, USA) at 30°C, during which luminescence emission (RLU) per OD_600_ was measured every 20 min with shaking prior to each read. Each assay was replicated on four separate occasions.

To estimate transcriptional efficiency, the mean light produced per bacterial cell was calculated at each time point by dividing the luminescence of each well (in RLU) by its absorbance (OD_600_). The maximum gradient of each RLU/OD_600_ curve was then calculated using nine consecutive data points during the early exponential phase. Within each assay, the relative *lux* expression rate in each *rpoB* mutant strain was obtained by normalizing each gradient to that of the ancestral strain control in the same plate. This provides a measure of the maximum rate of transcription per mutant.

## 3. Results

### (a) Fitness costs of rifampicin resistance across species of *Pseudomonas*

**Figure 1.**
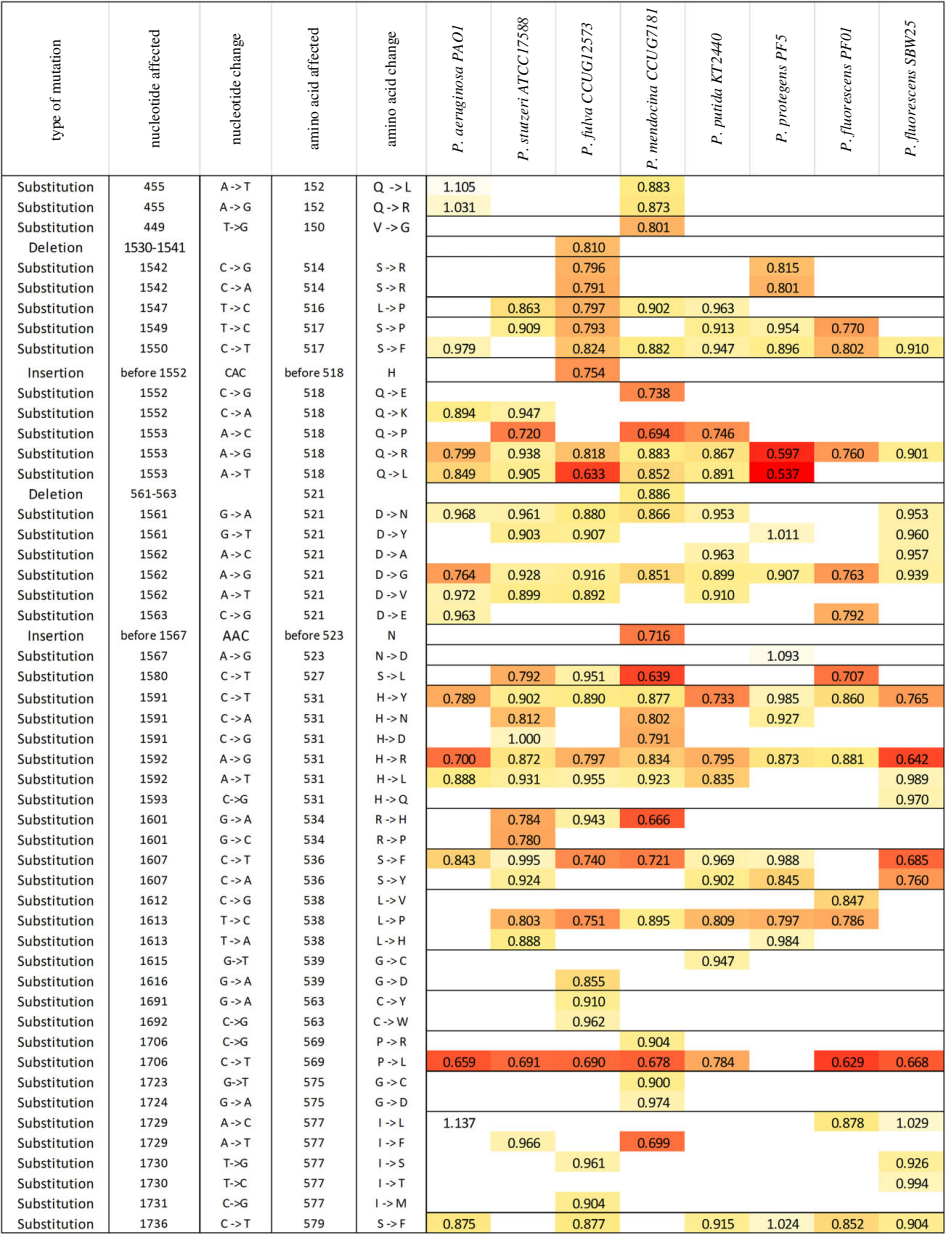
Fitness effects of resistance mutations across species*.* Numbers show the fitness of rifampicin-resistant mutants relative to their respective ancestral strains in the absence of antibiotics, such that a value of 1 represents equal fitness. Each competition assay was carried out with sixfold replication and the colours highlight variation in fitness, ranging from red for very costly to white for beneficial. (Online version in colour; in printed version, intensity of shdading indicates the cost of mutation.)

To estimate the relative importance of genetic background in determining the fitness cost of rifampicin resistance, we performed competitive fitness assays on a previously generated collection of rifampicin-resistant mutations from eight strains of *Pseudomonas.* Each of the competition experiments for the 161 unique strain-by-mutation combinations were replicated six times, giving 966 independent fitness measurements (figure 1). As shown, there is considerable variation in the cost of resistance. Owing to the low number of mutations common to all strains, as well as the high number of mutations found in only one strain, we analysed these data using a model consisting of just the main effects of strain and mutation. We find that although both factors significantly affect the cost of resistance (strain: *F*_7,876_ = 8.79, *p* < 0.001; mutation: *F*_51,876_ = 8.34, *p* < 0.001), mutation explains a greater proportion of the variation in fitness (mutation = 27.1% of variance; strain = 4.0% of variance). However, even if there is greater variance between mutations than strains, genetic background could still be affecting the cost of resistance. To test how commonly the cost of a mutation depended on its genetic background, we performed Bonferroni-corrected one-way ANOVAs on every mutation which arose in at least two backgrounds. Of these 33 comparisons, 14 (42.2%) are significant at the Bonferroni-corrected *p*-value of 0.0015, demonstrating that genetic background impacts fitness for almost half of these mutations under a conservative statistical test.

**Figure 2.**
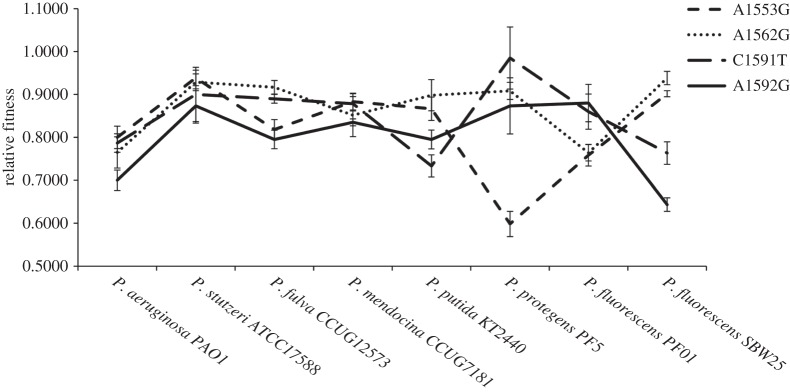
Epistastic effects of rifampicin resistance mutations on fitness across *Pseudomonas*. The fitness effect of the four common rifampicin resistance mutations across eight strains of *Pseudomonas* (±s.e.m; *n* = 6) is shown. The fitness of each resistant mutant was measured relative to its ancestor in the absence of antibiotics, such that a value of 1 represents equal fitness.

An alternative approach to estimate the effects of epistasis is to limit the analysis to just the four mutations common to all eight strains (figure 2). Although this limits the scope of the analysis, this approach makes it possible to estimate the contribution of the interaction between mutation and genetic background to the cost of a mutation. This analysis reveals significant effects of mutation (*F*_3,158_ = 7.53, *p* < 0.001) and genetic backgrounds (*F*_7,158_ = 7.13, *p* < 0.001) on fitness, and an interaction between mutation and genetic background (*F*_21,158_ = 7.37, *p* < 0.001). However, the proportion of variance explained by mutation is small (5.83%), whereas the explanatory power of genetic background is not much greater (12.88%). In contrast, the interaction term explains 39.92% of the variance. The implication of this result is that the interaction between a rifampicin resistance mutation and the genetic background plays a dominant role in determining the cost of resistance. Intriguingly, this epistatic interaction is driven by a complex interplay between mutations and genetic backgrounds, and not because all mutations are more costly in some strains than others.

### (b) Phylogeny and the costs of a mutation

**Figure 3.**
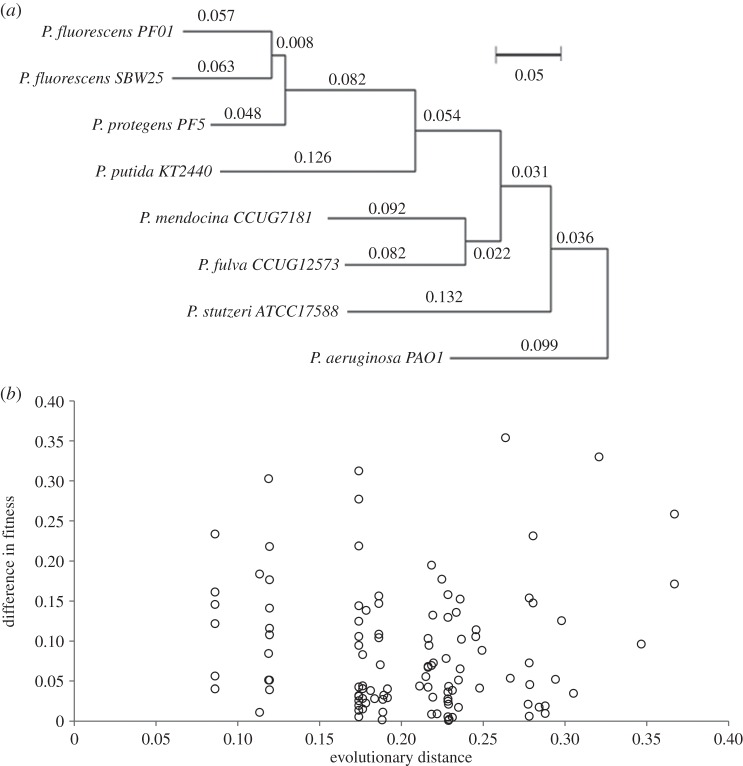
Evolutionary divergence does not predict epistasis. (*a*) Phylogeny of the eight ancestral strains of *Pseudomonas*. Branch lengths are proportional to evolutionary distance. (*b*) The contrast in the fitness effect of rifampicin resistance mutations between tips or nodes on the phylogeny shown as a function of evolutionary distance. We find no evidence that rifampicin resistance mutations have more divergent effects on fitness between distantly related bacterial strains than between closely related strains.

One important assumption of the above analyses is that they do not take phylogeny into account, and this may be an important bias. For example, intuition suggests that a mutation is more likely to have more similar effects on fitness in two closely related strains than in two distantly related strains. To test the hypothesis that phylogenetic distance drives epistasis, we tested for a positive correlation between genetic distance and epistasis using methods based on phylogenetically independent contrasts (PICs). As in PICs, we calculated the difference between neighbouring tips or nodes, thereby controlling for unequal relatedness between differing species (figure 3*a*). However, rather than standardize these contrasts by dividing by the underlying genetic distance, we looked for a relationship between genetic distance and the size of a contrast. If a mutation was found in all eight strains, it is possible to make seven contrasts. Because not all mutations were found in all strains, from the 33 mutations which were found in more than one strain it is possible to make 109 contrasts (figure 3*b*). We found no significant relationship between evolutionary distance and epistasis (Pearson's correlation: *r* = −0.480, d.f. = 107, *p* = 0.878). To see if evolutionary distance increases the probability of epistasis for any individual mutations, we re-analysed the data for the four mutations common to all strains, as these would have the most statistical power. None of the four show a significant correlation between evolutionary distance and epistasis (A1553G: *r* = −0.480, d.f. = 5, *p* = 0.275; A1562G: *r* = −0.379, d.f. = 5, *p* = 0.402; C1591T: *r* = −0.449, d.f. = 5, *p* = 0.312; A1592G: *r* = −0.619, d.f. = 5, *p* = 0.138). In short, mutations do not appear to have similar effects on fitness between more closely related strains. Therefore, at this scale, it appears that phylogenetic relatedness is not an important determinant of the probability of epistasis.

### (c) Molecular mechanisms underpinning cross-strain epistasis

Previous work has shown that compromised transcriptional efficiency explains much of the variation in the fitness cost of rifampicin resistance in *P. aeruginosa* [20], providing a potential molecular mechanism to explore the cost of resistance across strains. To link transcriptional efficiency with fitness, we transformed clones of *P. aeruginosa PAO1*, *P. putida KT2440* and *P. fulva CCUG12573* with a chromosomally integrated reporter that measures transcriptional efficiency. These strains were selected owing to their ease of genetic transformation, and the same seven *rpoB* mutations were transformed in each strain.

**Figure 4.**
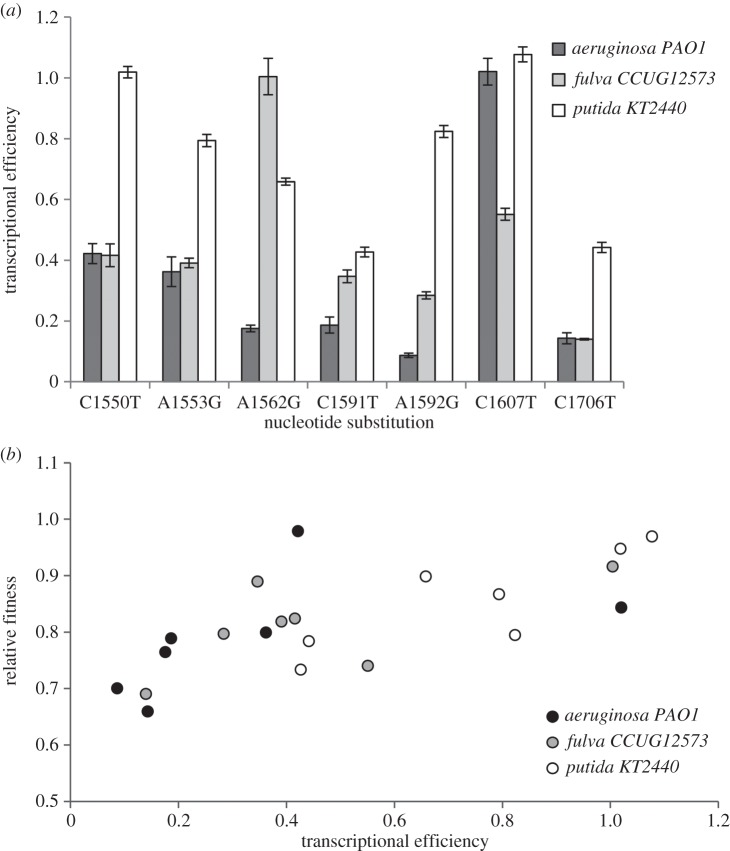
Linking epistasis to transcriptional efficiency. (*a*) The transcriptional efficiency (±s.e.m; *n* = 6) of seven rifampicin resistance mutations in *P. putida* *KT2440*, *P. aeruginosa* *PAO1* and *P. fulva CCUG12573*. (*b*) The correlation between relative fitness and transcriptional efficiency for the seven mutations common to these three strains. Transcriptional efficiency and fitness were independently assayed relative to the ancestral strains, such that a value of 1 denotes fitness or transcriptional efficiency equal to the ancestor.

Given that transcriptional efficiency is likely to be an important determinant of fitness across strains, two mechanisms could explain why resistance mutations have background-dependent effects on fitness. First, it is possible that the same mutation has different effects on transcriptional efficiency in different strains. Consistent with this mechanism, we find transcriptional efficiency is significantly affected by mutation (figure 4*a*; *F*_6,63_ = 49.61, *p* < 0.001, variance explained = 37.5%), strain (figure 4*a*; *F*_2,63_ = 105.54, *p* < 0.001, variance explained = 26.6%) and, crucially, an interaction between strain and mutation (figure 4*a*; *F*_12,63_ = 18.48, *p* < 0.001, variance explained = 28.0%). In other words, the same rifampicin resistance mutation has different effects on transcriptional efficiency in different strains of *Pseudomonas*, resulting in epistatic interactions for fitness between resistance mutations and genetic background. Second, it is possible that even when resistance mutations have consistent effects on transcriptional efficiency across strains, compromised transcriptional efficiency carries a greater cost in some strains than others. To test if the relationship between transcription and fitness is strain-dependent, we analysed the fitness of these 21 transformed mutants using a general linear model with transcription rate fitted as a covariate and strain fitted as a fixed factor. As expected (figure 4*b*), we find that transcriptional efficiency significantly correlates with fitness (transcription: *F*_1,15_ = 12.3, *p* < 0.005, variance explained = 45.1%). This correlation is not significantly affected by strain (*F*_2,15_ = 0.45, *p* = 0.645, variance explained = 5.7%), nor by an interaction between strain and transcription rate (*F*_2,15_ = 0.37, *p* = 0.697, variance explained = 4.7%), demonstrating that the relationship between transcriptional efficiency and fitness is constant across strains.

## 4. Discussion

Although genetic background is increasingly being seen as an important influence on adaptation (reviewed in [24,25]), experimental estimates of its role remain rare. Here we demonstrate that epistatic interactions between antibiotic resistance mutations and the genetic backgrounds in which they evolve are key in determining the fitness costs of rifampicin resistance. Given that fitness costs have a central role in the long-term dynamics of resistance, genetic background is likely to play a key role in the evolution of resistance. Specifically, we find that the fitness effects of over 40% of rifampicin resistance mutations differ significantly between species of *Pseudomonas.* In agreement with previous works [15,20], we find that compromised transcriptional efficiency plays a key role in determining the cost of rifampicin resistance. Moreover, the relationship between transcriptional efficiency and fitness is constant across strains, and the epistatic effects of rifampicin resistance on mutations arise from the fact that mutations have background-specific effects on transcriptional efficiency. In other words, in this system the relationship between phenotype (transcriptional efficiency) and fitness is constant over the range of tested values, but the phenotypic effect of mutations is complex and strain-specific.

One feature of the design of our study is that we tested for background effects at quite a broad phylogenetic scale. *Pseudomonas* is a genus that is well known for its remarkable phenotypic and genetic diversity [14]. The strains used in this study were selected to sample a broad range of this diversity without sacrificing the experimental tractability that is required to accurately measure the fitness costs of resistance. From our data, it is clear that genetic background has a pervasive impact on the cost of resistance at this phylogenetic scale. However, our study does not provide any insights into whether this is likely to be the case at a finer phylogenetic scale, such as different clones from the same species. Although there is almost certainly less diversity within any one species than observed between our isolates from many different species, this is not to say there is considerable genomic diversity within species of bacteria [26–28]. Equally, the cost of resistance has been shown to be epistatic even when strains only vary by as little as a single mutation [11,12]. Taken together, there is no reason why genetic background effects cannot also be as pronounced as observed here at considerably smaller evolutionary scales.

The cost of antibiotic resistance is predicted to be crucial to the clinical evolutionary dynamics of resistance [1,2]. As genetic background strongly affects the cost of resistance, this may explain why certain lineages of pathogen species are more likely to be epidemic than others [29]. The explanation for these so-called dominant or epidemic strains is often the possession of specific traits, such as particular antibiotic resistance determinants or virulence factors. But this leads to the question of why these strains have successfully acquired these traits, but other clones have not. Although there are several potential reasons, one interpretation is that these genetic backgrounds are the ones where these traits possess the lowest cost. This raises the important point that the function and cost of clinically important traits measured on a particular (often laboratory-adapted) strain should be assumed to be identical for all strains and clones.

In our experiment, we investigated only the relationship between fitness and transcription in a single environment. However, not only do clinical populations of bacteria encounter many different environments, but the cost of antibiotic resistance has been shown to be environment-dependent [30]. It is unlikely that transcription will be equally correlated with fitness across all possible environments. Indeed, it has been previously shown that the costs of *rpoB* mutations are reduced when the need for transcription is artificially lowered [31]. Therefore, in some environments (or rather in some combinations of environments and genetic backgrounds) the relationship between transcription and fitness will disappear. However, the relationship between transcription and fitness in chronic *Mycobacterium tuberculosis* infections is likely to be strong. Using rifampicin to treat *M. tuberculosis* is rifampicin's most common clinical use, and consequently many *M. tuberculosis* populations evolve resistance to rifampicin via mutations in *rpoB* [6,32]. These populations will often subsequently adapt to the costs of *rpoB* mutations by fixing compensatory mutations [32], which in other systems have been shown to restore fitness by directly restoring transcriptional efficiency [15,33]. Therefore, it would appear that transcriptional efficiency is also likely to be tightly correlated with fitness in clinical *M. tuberculosis* infections.

Additionally, as genetic background can significantly affect the cost of resistance, this could have important consequences for adaptation to the cost of resistance. Compensatory adaptation to the cost of antibiotic resistance is predicted to be critical to its evolutionary dynamics [2], as it can slow or even prevent the extinction of resistant strains in the absence of selection for resistance. As the probability of compensatory adaptation should depend on the cost of a mutation, genetic background therefore has a central role in compensatory adaptation to the cost of resistance. Additional support for this comes from rifampicin-resistant *M. tuberculosis*, which is one of the best-characterized examples of compensatory adaptation [32]. It has been demonstrated that some strains can compensate very rapidly over the course of a few weeks. However, other strains, possessing the exact same mutation, took more than 2 years to compensate for this mutation, whereas yet other strains did not show compensatory dynamics at all [32]. Admittedly, there are other explanations for this besides genetic background, such as adaptation to the infection environment rather than to compensation *per se.* However, it could also be that compensation is more rapid on genetic backgrounds in which the mutation possesses the greatest cost.

Given that *rpoB* is highly conserved, it is surprising that the same mutation has different effects on transcriptional efficiency in different strains of *Pseudomonas*. However, this is overlooking the many different and often less conserved cellular systems involved in transcriptional efficiency. In support of this argument, previous work has shown that *rpoB* mutations can have highly pleiotropic effects [34–36], including altered expression of *rpoC* [20], another subunit of RNA polymerase. Therefore, alterations to the expression of other genes involved in transcription may explain the variable impacts of these mutations on transcriptional efficiency, assuming the regulation of these genes is less conserved between species than the structure of *rpoB*. However, we stress that this is quite a speculative point, and further molecular work beyond the scope of this study would be required to test this hypothesis rigorously.

In summary, we present evidence that genetic background is a key determinant of the fitness costs of antibiotic resistance. As such, the effect of genetic background should not be ignored when studying the evolution and epidemiology of bacterial pathogens.

## Data accessibility

Data are available from Dryad (doi:10.5061/dryad.qn370).
